# Short and Long-Term Attentional Firing Rates Can Be Explained by ST-Neuron Dynamics

**DOI:** 10.3389/fnins.2018.00123

**Published:** 2018-03-02

**Authors:** Oscar J. Avella Gonzalez, John K. Tsotsos

**Affiliations:** ^1^Department of Electrical Engineering and Computer Science, York University, Toronto, ON, Canada; ^2^Laboratory for Active and Attentive Vision, Centre for Vision Research, York University, Toronto, ON, Canada

**Keywords:** visual attention, single cell, ST-neuron, firing rate, neural selectivity

## Abstract

Attention modulates neural selectivity and optimizes the allocation of cortical resources during visual tasks. A large number of experimental studies in primates and humans provide ample evidence. As an underlying principle of visual attention, some theoretical models suggested the existence of a gain element that enhances contrast of the attended stimuli. In contrast, the Selective Tuning model of attention (ST) proposes an attentional mechanism based on suppression of irrelevant signals. In this paper, we present an updated characterization of the ST-neuron proposed by the Selective Tuning model, and suggest that the inclusion of adaptation currents (Ih) to ST-neurons may explain the temporal profiles of the firing rates recorded in single V4 cells during attentional tasks. Furthermore, using the model we show that the interaction between stimulus-selectivity of a neuron and attention shapes the profile of the firing rate, and is enough to explain its fast modulation and other discontinuities observed, when the neuron responds to a sudden switch of stimulus, or when one stimulus is added to another during a visual task.

## Introduction

Attention can be widely defined as “the selective prioritization of the neural representations that are most relevant to one's current behavioral goal” (Buschman and Kastner, 2015). Since James' pioneering work (James, 1891), research on attention has aimed to discover a precise and systematic description of how the brain is able to manage its limited resources for performing complex cognitive and behavioral tasks. Visual attention, as one component of attention, has received significant interest (Itti et al., 2005; Carrasco, 2011; Posner, 2011), leading to the proposal of detailed descriptions of aspects like bottom-up attention (Itti and Koch, 2001; Rutishauser et al., 2004; Itti, 2005) and top-down control (Corbetta and Shulman, 2002; Oliva et al., 2003; Buschman and Miller, 2007; Bressler et al., 2008), signal integration (Corbetta et al., 1991; Rao et al., 1997; Eagleman and Sejnowski, 2000), or focus of attention (Koch and Ullman, 1987; Desimone and Duncan, 1995; Tsotsos et al., 1995).

Mathematical models as a wide-spread strategy are used to make insightful predictions about neural communication, and brain dynamics in general (Hodgkin and Huxley, 1952; Destexhe et al., 1998; Kandel et al., 2000; Dayan and Abbott, 2001; Shriki et al., 2003; Izhikevich, 2004). Concerning visual attention, a number of relevant models have been proposed to study particular aspects concerned with the way single neurons and circuits process incoming information during visual tasks (Tsotsos, 1990; Niebur and Koch, 1994; Reynolds et al., 1999; Deco and Lee, 2002; Reynolds and Heeger, 2009); One of these aspects, treated by different studies and that currently draws special interest, is the mechanism neurons use during attentional tasks to accurately encode, classify and prioritize dissimilar information using only their firing rates. For instance, in the biased competition model by Reynolds et al. (1999), stimuli compete for a cortical representation, and the average firing rate (response) of a neural population depends on the interaction between the selectivity of the cells for one particular type of stimulus or feature, and the modulation induced by attention. The feature similarity model of Martinez-Trujillo and Treue proposes that attention enhances neural selectivity (Martinez-Trujillo and Treue, 2004), thus causing neurons to increase their firing rate. The idea aligns well with the normalization model (Lee and Maunsell, 2009; Reynolds and Heeger, 2009) in which such enhancement relates to the contrast between the attended stimulus and the surrounding background perceived by a neural population. Other models also explore the relation between the detailed anatomy of the neurons and the response to the attentive signal. The Feedback model for example, acknowledges attention as a top-down process that operates via cortical feedback, and represents it using a gain factor that modulates the activity of impinging connections to a given neuron (Spratling and Johnson, 2004). It also takes into consideration physiological properties such as the roles of the basal (feedforward) and apical (feedback) connections, and how by adding those elements it is possible to resemble the response of pyramidal cells during attentional tasks (Spratling and Johnson, 2004). In the Selective Tuning model (ST) (Tsotsos, 1990, 2011; Rothenstein and Tsotsos, 2014), attention is also embodied as a top-down signal; but in contrast to other models, its selection mechanism fully relies on suppression of the irrelevant inputs to each neuron instead of the enhancement of their activity (Tsotsos, 1990, 2011), as supported by strong experimental evidence (Cutzu and Tsotsos, 2003; Loach et al., 2005; Hopf et al., 2006; Bartsch et al., 2017).

Adaptation mechanisms are well known for their facilitating role in detecting weak signals by means of stochastic resonance (Wiesenfeld and Moss, 1995) or through sub-threshold oscillations enhancement (Dorval and White, 2005). In a previous modeling study Rothenstein and Tsotsos (2014) found that by incorporating adaptation mechanisms, the overall performance of the ST neuron was improved during a simple attentional task. Thus, counterbalancing the rapid saturation of the firing rate due to the presentation of a highly affine stimulus, while resembling the shape of the firing profiles recorded in V4 visual cells (Kosai et al., 2014) (Figures 2, 3 therein). As a follow up of that study, in the present paper we perform a detailed characterization of the ST-neuron firing pattern with and without adaptation currents (Ih) (Pape, 1996). Next, and following the design by Reynolds et al., (Reynolds et al., 1999) we implement a simple circuit to explore various scenarios in which adaptation currents play a role in reshaping the firing profile of the neuron, either by fine-tuning it, or by increasing the sensitivity of the cell to the attentional signal.

The contribution of adaptation currents to the cell's dynamics is further highlighted, by simulating a set of experiments that strikingly uncovers the interplay between neural selectivity and attention as a twofold effect. It first creates a transitory and a stationary scenario in the firing response of the recorded cell; and second, induces the transition between the firing patterns evoked by two competitive stimuli in a task-dependent fashion. We also compare the results of our simulations against experimental findings, and show how the incorporation of Ih on the ST-model leads the response to closely resemble the transient and long-lasting effects observed in experimental data.

## Methods

Our model consists of four essential elements: the ST-neuron model, the circuit's design and connectivity, the neural selectivity, and the selection mechanism of attention.

### The ST-neuron

The Selective Tuning model of attention (ST) relies on the ST-neuron as its building block (Tsotsos, 1990, 2011; Tsotsos et al., 1995). The ST-neuron is responsible for the integration and propagation of signals across the visual hierarchy, and both implements attentional selection as well as displays modulations resulting from top-down attentional signals. As a rate-based model, the response is quantified by the temporal evolution of the firing rate (FR) according to Equation (1):

(1) $$\begin{array}{r} \frac{dFR}{dt}=\frac{1}{\tau }\cdot \left(-FR+S\left(P\right)\right) \end{array}$$

In this expression, *P* is the synaptic input, $S\left(P\right)=\frac{MP^{\xi }}{\sigma^{\xi }+P^{\xi }}$ is the Naka-Rushton sigmoid function, whose value depends on the maximum firing rate *M*, the semi-saturation constant σ, i.e., the particular value of the input for which S(σ) = $\frac{1}{2}M$, and the constant factor ξ that determines the slope of S(P), i.e., how quickly it saturates. Aiming to resemble the time evolution of the firing rate FR, the response of the cells was restricted to the interval [0,1] by setting *M* = 1, and the semi-saturation constant σ = σ_0_, with σ_0_ = 0.25·M. The latter was chosen in order to prevent P from growing too fast and to avoid step-wise behavior of the activation function. The factor ξ = 3, is a heuristic parameter whose value for neurons in the visual cortex was previously reported by Wilson (1999). With this choice of values for all parameters we ensure that for *P* = 1, $S\left(P\right)=\frac{M}{{0.25\cdot M}^{\xi }+1\;}≅\;0.98$; i.e. the reachable ceiling of the rate is not significantly attenuated irrespective of M (see **Figure 2A**). This represents a normalized and ideal scenario in which all impinging connections to a neuron are excitatory. Finally, τ represents the time constant of the activation and was set to τ = 10 ms, thus satisfying the kinetics of gabaergic receptors such as GABAA, and matching the average duration of the post–inhibition refractory period (Whittington et al., 2000; van Aerde et al., 2009).

Similar to Rothenstein and Tsotsos (2014), we considered the effect of adaptation currents Ih on the ST-neuron, and incorporated them in the dynamic equation as additive factors that modulate the magnitude of the semi-saturation constant σ. The new σ(t) is then re-computed at every time-step using Equation (2) as follows:

(2) $$\begin{array}{r} \sigma \left(t\right)=\sigma_{0}+f_{slow}\cdot H_{slow}\left(t\right)+f_{fast}\cdot H_{fast}\left(t\right) \end{array}$$

where σ_0_ is the original parameter. Adaptation currents consist of two different components *H*_*slow*_ and *H*_*fast*_, each evolving within a particular time-scale, coupled to the value of the firing rate FR, and whose time course is scaled by the characteristic time constant τ_x_ with x being either *fast* or *slow*. In turn, *f*_*slow*_ and *f*_*fast*_ are the values of the amplitude for each contribution. The temporal evolution of the two components is given by the Equation (3):

(3) $$\begin{array}{l} \frac{{dH\left(t\right)}_{fast}}{dt}=\frac{1}{\tau_{fast}}\cdot \left(-{H\left(t\right)}_{fast}+FR\left(t\right)\right)\\ \text{ and}\\ \frac{{dH\left(t\right)}_{slow}}{dt}=\frac{1}{\tau_{slow}}\cdot \left(-{H\left(t\right)}_{slow}+FR\left(t\right)\right) \end{array}$$

Equations (1–3) are independently updated for each neuron at every time step (Δt = 2 ms) using a customized Runge-Kutta 4 algorithm implemented in MATLAB 2016a, (The MathWorks, Inc.). The original details of the implementation can be found in Wilson (1999).

### Circuit design and connectivity

**Figure 1:**
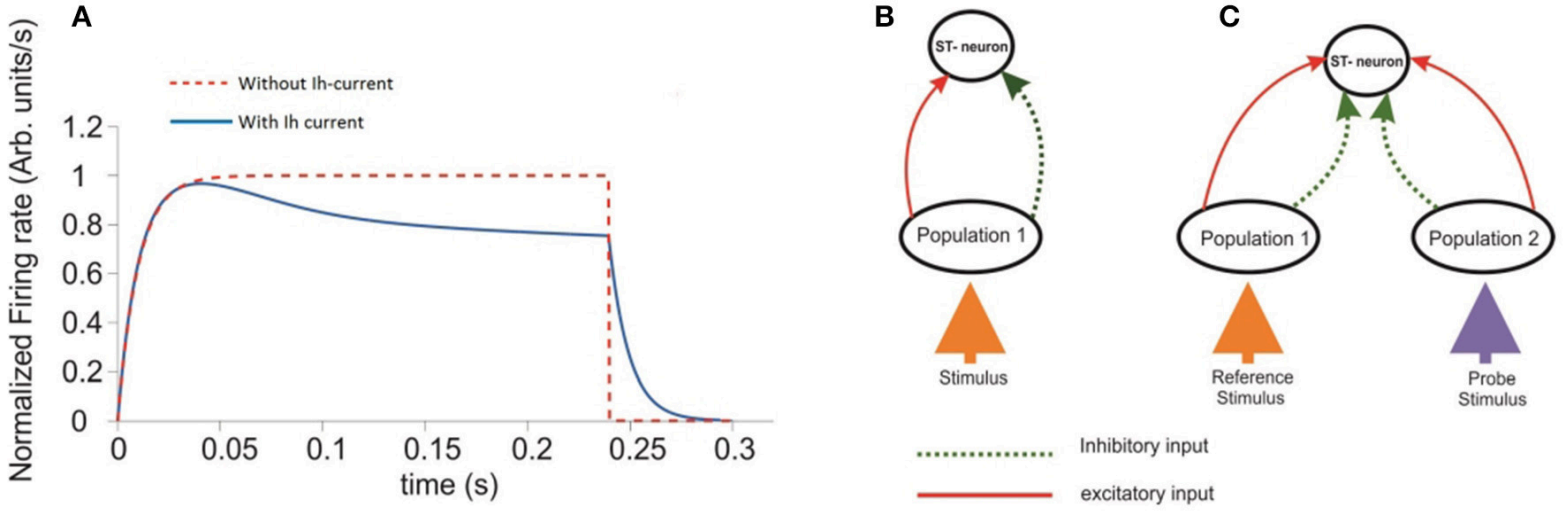
Response of a single ST-Neuron to a fully preferred stimulus. **(A)** Activation of the neuron occurs after presenting the stimulus during a simulated attentional task with 300 ms total elapsed time. The red curve corresponds to the ST-neuron activation in the absence of the adaptation mechanism (Ih). The blue curve represents the same dynamics, when Ih currents are incorporated. **(B)** Schematic representation of the minimum circuit used to study the selectivity and attention aspects on the ST-neuron's response. The top unit represents a cell with a highly complex receptive field able to process abstract object representations. The unit at the bottom represents the average response of neurons selective for that stimulus. **(C)** An extended representation of the circuit used to model selection and attention. The diagram extends the circuit shown in **(B)**. Where each population (ellipses) has high selectivity for one of two incoming stimuli represented by the colored arrows.

Following the original design by Reynolds et al. (1999), our circuit aims to represent a three tier structure, in which the response of the top-most unit quantifies the model's performance. The time course of this response was computed when the representations of two stimuli, each of which could be located either within or outside the cell's receptive field (RF), competed for representation (see Figure 1C). The bottom layer represented by two colored upwards arrows, contains the input representation. The Intermediate layer consists of two units, each accounting for the average response of individual populations (black ellipses) of ST-neurons, and are tuned to the stimulus directly below them. This level represents the activation of the populations at V1-V2 cortices. In turn, the neuron located at the top was defined as the main neuron (top circle). This unit represents a V4 cell, whose complex receptive field is able to process whole object representations.

Inputs at the bottom are represented by particular combinations of excitatory and inhibitory connection weights projected to the intermediate layer. Each intermediate population receives excitatory (red continuous arrows) and inhibitory connections (green dotted arrows) from the input, and project them to the top. The top unit receives both types of feed-forward inputs from the intermediate layer. Figure 1B shows a simplified version of the circuit in which a single stimulus is presented and processed. Connection weights were defined in the interval [−1, 1], with the convention that w is inhibitory if −1 ≤ w < 0, and excitatory if 0 ≤ w ≤ 1. In consequence, any potential changes to the stimulus properties should be reflected as changes in the combination of connection weights representing it. During the time course of each simulation the set of excitatory and inhibitory connection weights from the intermediate layer onto the target (top) neuron remained fixed. Consistent with our assumptions, the representation of a given stimulus consisted of setting only the excitatory and inhibitory connection weights from the bottom to the intermediate layer. All other parameters were fixed within and across simulations, unless otherwise stated.

### Neural selectivity

Neural selectivity is the mechanism by which a neuron raises its firing rate when a stimulus has a certain feature matching its tuning curve. Thus, a preferred stimulus is one for which the neural selectivity is high. In order to incorporate selectivity into the circuit, and provided that neurons were connected through inhibitory and excitatory inputs with particular connection weights, we assumed for a preferred stimulus an excitatory (E) connection weight *w*_*E*_ belonging to the interval 0.75 < *w*_*E*_ ≤ 1, and consequently an inhibitory (I) weight *w*_*I*_ = 1 − *w*_*E*_, belonging to 0 ≤ w_*I*_ ≤ 0.25. In the case of a stimulus with low selectivity i.e., one for which the cell selectivity is low, the inhibitory weight approached *w*_*I*_ = 1 and the excitatory *w*_*E*_ = 0. For the sake of convenience, and bearing in mind that for the current normalized case the sum of weighted E and I inputs satisfies ∑|*w*_*I*_| · *I* + |*w*_*E*_| · *E* = 1, any stimuli with 0.7 ≤ *w*_*E*_ ≤ 0.75 were considered as of neutral selectivity. Stimuli with 0.75 ≤ w_*E*_ ≤ 1 were defined as preferred (or having high selectivity), and stimuli with 0.5 < *w*_*E*_ < 0.7 were defined as non-preferred (or having low selectivity).

### ST's top-down attentional signal

The attentional signal was implemented in consonance with the ST model, by creating a top-down branch-and-bound selection mechanism that picked the targets and suppressed the neural representation of the distractors, as described in Tsotsos (2011). The amplitude of the signal between belonged to the range [0, 1], and was computed like the absolute difference between the magnitude of the activation of the intermediate units, and the resulting factor was used to multiply the weights of the unit, associated to irrelevant input. This process has been fully described several times previously, most recently in Tsotsos (2011) and thus will not be repeated here.

## Results

### Characterizing the ST neuron dynamics

In order to extend previous findings, we first characterized the time course of the neuron in relation to basic parameters, and then by modeling the response of the neuron after incorporating adaptation mechanisms, we evaluated their effect on the cell's firing dynamics during a set of simulated visual tasks.

In absence of adaptation mechanisms the activation of the ST-model neuron is determined by the two parameters σ and τ of the Naka-Rushton function (see equation 1. in section Methods). Although this function was first introduced in order to account for the adaptive saturation of photoreceptors to particular illumination conditions, its role in shaping the response of the ST-neuron was not previously addressed.

**Figure 2:**
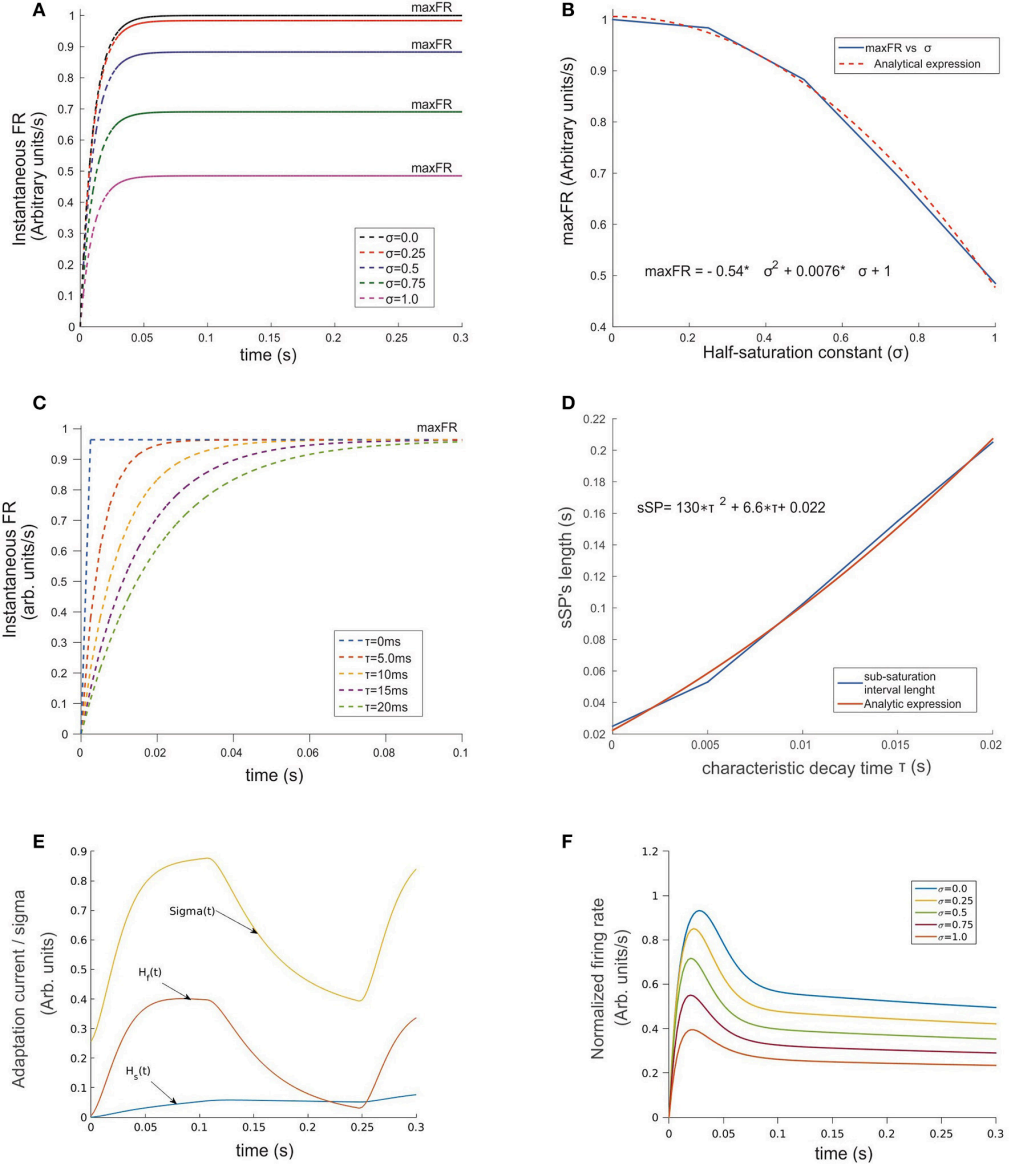
Temporal evolution of the ST-neuron's firing rate. **(A)** For a constant input, the amplitude of the firing rate has a transitory pre-saturation period which is independent of the half saturation constant σ. However, after this point and depending on its magnitude, increasing σ led in a minor or major proportion the saturation rate of the cell to fall and reach smaller maxFRs. **(B)** Analytical expression of the relation between variables depicted in **(A)** firing rate and σ are related through a quadratic function for which small values of σ near 0 rapidly makes maxFR ≅ M. **(C)** A similar relation rules the effect of τ on the time required by the firing rate to saturate when σ was kept fixed. The simulation shows strong modulation before the 100 ms point of each simulation. In spite of maxFR remaining unchanged, the duration of the sub-saturation period increased proportionally to τ following the trend plotted in **(D)**. A representation of the temporal pattern for the fast (*H*_*f*_) and slow (*H*_*s*_) components of the Ih-current is shown in **(E)**. The combined effect of the two components modulates the firing rate by adding temporal dependence to σ (see Equation 2 section Methods), whose dynamics is represented by the top trace in **(E)**. The response of the top cell in **(F)** shows the effect on the FR-profile when submitted to the action of the synaptic inputs and the activation of Ih. Here the values of σ are identical to **(A)**. Note in the latter the decaying post-saturation profile and the generation of bumps before reaching the stationary firing regime.

As a two-step exercise we first fixed the value of τ and varied σ and then we flipped this, fixing σ while varying τ. In the first case, we assumed *M* = 1.0, and σ = k·M, for *k* = 0, 0.25, 0.5, 0.75, and 1.0, obtaining the response curve shown in Figure 2A. Its shape followed a sigmoid pattern with amplitude of saturation (maxFR) proportional to the choice for σ, counterbalanced by P, and scaled by M (red curve in Figure 1A). Our simulations show that for every σ, the FR-profile saturated within the initial 50 ms. In the case of larger σ, any variation in k led to monotonic decrements of the saturation rate's magnitude (maxFR) (Figures 2A,B). The analytical relation was well described by the expression *maxFR* = −0.54 · σ^2^ + 0.0076 · σ, with a resulting norm of residuals nr = 0.024696. This result suggests that in the limiting condition σ → 0, the smaller the value of σ the closer maxFR is to *M*.

By fixing σ and varying τ within a biologically plausible range with τ = 0.0, 5.0, 10.0, 15.0, and 20.0 ms rather than variations on maxFR, we observed significant effects on the timing required by the sub-saturation period (rising phase) to reach maxFR (see Figures 2C,D). In spite of the reasonable behavior of the model's output for τ ≅ 10–20 ms, we embraced experimental observations from previous studies (Jensen et al., 2005) choosing τ = 10 ms, which on one hand accounts for an acceptable durations of the sub-saturation period of around 20 ms, and on the other coincides with the reported time constant of GABAergic synapses such as GABAA, aligning also with the idea that “.tonic inhibition in single neurons increases the firing threshold and reduces the membrane time constant …” (Hutt, 2012). In the case of τ shorter than 10 ms unrealistically fast saturation of the rate occurred, while for τ much larger than 20 ms, sub-saturation intervals were also extremely long. In general, the response of the model shows consistency with experimental findings (Kandel et al., 2000) deploying a relation between the duration of the time required for the firing rate to saturate, i.e., the sub-saturation period sSP and τ, given by the analytical expression *sSP* = 130 · τ^2^ + 6.6 · τ + 0.022, with a norm of residuals *n* = 0.00775. Although the results for smaller τ's might reflect the action of other mechanisms, those do not necessarily represent the dynamics in the visual cortex (Cavelier et al., 2005).

A general result extracted from this simple analysis shows that far from interfering with one another, σ and τ control and modulate different parameters of the cell's activation, and their joint action reliably accounts for the efficacy of individual neurons to tune their firing to particular feature(s) of the synaptic representation of a certain stimulus.

### Effects of the adaptation currents (Ih) on the firing rate of a single cell

An overall comparison between the FR-profile of the neuron without Ih and with Ih is depicted in Figure 1A. The stimulus onset occurred at *t* = 0 and the removal at *t* = 250 ms. Note the unaffected FR-profile's rising phase of the with-Ih scenario (blue trace) and the appreciable changes occurring during the post-saturation of the with-Ih case compared to the non-Ih case (red trace). As in Rothenstein and Tsotsos (2014) Ih currents are represented by the linear combination of a slow (*H*_*s*_) and a fast (*H*_*f*_) component, whose time courses are depicted in Figure 2E by the blue and purple traces respectively. The modulation imposed on the constant σ (yellow trace on top) shows a periodic signal that slowly raises from σ_0_ to its maximum within ~130 ms, and exponentially decays within a comparable interval (~120 ms). As previously mentioned, the FR's rising phase remains unaffected and the overall effect is constrained to its post-saturation phase in a two step process (see Figures 2E,F): In the first, during a transitory interval (~50 ms), the firing rate is driven by the activation of the Ih's fast component H_*fast*_, leading the FR-profile to rapidly decay to ~70–80% of its maximum (maxFR). In the second, and due to H_*fast*_ having reached its maximum, the slow activation of H_*slow*_ takes over the control and reduces the speed of the FR decay, leading to a pseudo-plateau in the FR-profile, in which, in absence of any further changes in the stimulus, the FR remains constant.

### Response of ST-neuron (with Ih) to stimuli with different selectivity

To run this set of experiments we initially assumed attention not to be directed to the stimuli; thus the time course of the FR-profile only depended on the neuron's selectivity to a given stimulus. We simulated various (uniquely defined) types of inputs with selectivity being accounted for by the relative contribution of the inhibitory and excitatory connections.

In each experiment a given pair of stimuli was shown as input to the circuit of Figure 1C (for details see section Methods). To maintain consistency with psychophysical studies, we refer to the first stimulus as the reference, whose onset time occurred at *t* = 0 ms and its removal at *t* = t′ with t′ > 0, coinciding with the onset of the second stimulus that remained active until the end of the simulation and was denoted by the probe. The time *t* = t′ was designated as the switching time. In addition, the processing of each stimulus activated only one of the intermediate populations, and the probe stayed active until the end of the simulation, whose total duration of 300 ms was considered to be long enough to allow input-related information to propagate from the bottom to the top neuron (target).

**Figure 3:**
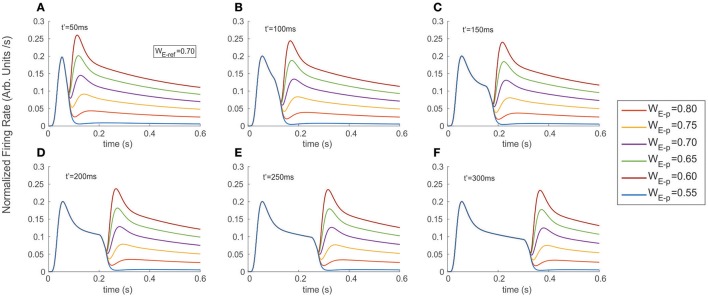
Stimulus exchange leads to strong discontinuities and transients in the FR-profile. Experiments were run simulating a fixed interval of 600 ms, and exchanging the stimulus at **(A)** t′ = 50 ms, **(B)** t′ = 100 ms, **(C)** t′ = 150 ms, **(D)** t′ = 200 ms, **(E)** t′ = 250 ms, and **(F)** t′ = 300 ms. Colored traces indicate the probe's selectivity characterized by the excitatory weight WE-p (refer to labels in Methods for details). Switching from a neutral reference (W_E−ref_ = 0.7) to a probe with larger or smaller selectivity created unstable surges of firing, followed by a stationary state. Note that in the case of a late t′, the transitory state did not interfere with the original time course of the FR-profile, but took place after the cell's recovery and near to the FR-profile's plateau.

Figure 3 shows the FR-profile's time course of the top neuron being initially driven by the reference, whose rising phase remained unaltered irrespective of how early t′ occurred, while being significantly affected on its post-saturation period in two ways. First, a latency appeared, caused by the decay of the initial FR and second, a sudden rebound appeared with maxFR depending on the probe alone. During the latency, and as an effect of switching inputs, the FR-profile became unstable leading to a transient drop and catch phase characterized by a discontinuous change of concavity and followed by a fast regain of firing. Once the FR surpassed maxFR due to the cell being engaged to the probe, the profile decays following the dynamics described in the previous section, with a pseudo-stationary state being ruled by the slow Ih's component. In every experiment a neutral reference i.e. excitatory synaptic weight W_E−ref_ = 0.7 (blue continuous trace) systematically preceded the probe, each of which had identical (W_E−p_ = 0.7), larger (W_E−p_ = 0.75, 0.80) or smaller selectivity (W_E−p_ = 0.65, 0.60, 0.55) than the reference. While the larger probes led to steeper jumps in the firing rate and bumps characterized by large maxFRs, stimuli with lower selectivity led to an even faster decay of the FR. The stationary response always equated the stationary response evoked by the probe in the absence of other inputs. Note that probes with identical selectivity to the reference did not align with the expected smooth profile evoked by the reference. An explanation to this is that the original tuning (i.e., the combination of weights) of the intermediate unit processing the probe was different from that of the reference and in consequence led to small bumps in the model (see purple traces in Figure 3) Measuring the plausibility of this effect needs further study and is left as an interesting open research point.

In general, the distortion in the reference's FR-profile was easier to recognize for probes presented briefly after the reference's onset i.e., t′ less than 200 ms. This result was consistent irrespective of the probe's selectivity (compare the shapes of the profiles in 3.A-3.C against those in 3.D-3.F). Note that in the case of a late t′, the transitory state did not interfere with the original time course of the FR-profile, but took place once the cell was close to the FR-profile's plateau, which could be interpreted as the replication of the original activity, but now due to the probe and with a different base rate.

Concerning the latency, our results show that for probes with less selectivity than the reference, the firing dropped and slowly recovered producing a smooth trough in the FR-profile, whose depth and width specifically depended on the relative difference of selectivity between both stimuli, being wider for less preferred probes, while in the case of probes with larger selectivity than the reference the width of the trough was negligible, and the FR-profile discontinuously lost and regained firing after switching stimuli. In general the particular shape and steepness of the bumps depended on the relative selectivity of the reference and probe, and once the transition occurred the rate slowly tended to stabilize around the stationary state evoked by the probe.

### Adding the probe to the reference modulates FR-profile but induces no latencies

**Figure 4:**
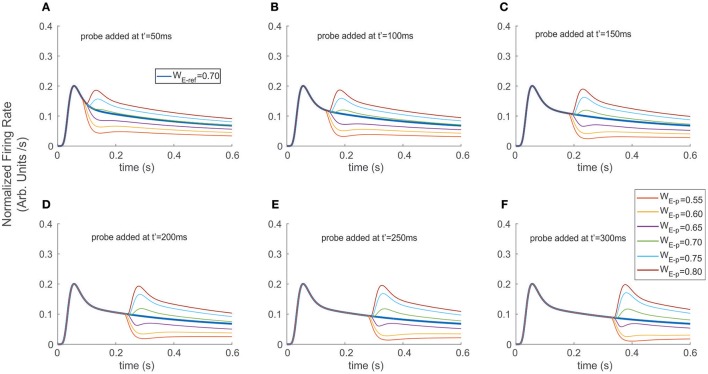
Adding a probe to the reference destabilized and induced transients on the firing rate. The reference stimulus was presented at *t* = 0 ms (W_E−ref_ = 0.7), and different probes were added at **(A)** t′ = 50 ms, **(B)** t′ = 100 ms, **(C)** t′ = 150 ms, **(D)** t′ = 200 ms, **(E)** t′ = 250 ms, and **(F)** t′ = 300 ms. Similar to the exchange experiment, transient bumps/troughs indicated sharp variations in the FR-profile. However, the shape and amplitude ratios between the principal and secondary peaks depended on the probe's addition time t′, for the case of probes with larger relative selectivity than the reference (see secondary bumps in **A–F**). In the case of probes with less selectivity, the transients exhibited variable concavities and lengths, thus led to cell responses with significantly reduced and more unstable firing rates (e.g., purple traces).

As a second scenario, instead of switching stimuli at t′, we modeled a condition in which the probe was added to the reference, while computing the time course of the top neuron's FR-profile (Figure 4). We ran the experiment for different probe selectivity and onset times t′ as follows: W_E−p_ = 0.55, 0. 60, 0.65, 0.70, 0.75, 0.80 (recalling that W_I−x_ = 1-W_E−x_ with *x* = ref or p), using a neutral reference (i.e., W_E−ref_ = 0.7) presented at *t* = 0. The FR-profiles in Figure 4 show that in contrast to the previous case (see Figure 3), and in the absence of attention, adding the probe at *t* = t′ produced no decaying latencies. Furthermore, probes with larger selectivity than the reference induced almost instantaneous rebounding bumps but in this case the amplitude of maxFR for the two stimuli never reached that of the reference alone, while less preferred probes led to a sudden drop followed by a less frequent but sustained and regular firing of the cell. Without exception for all probes, the value of maxFR was fixed across each of the diagram showing t′ = 50, 100, 150 ms. In contrast, for t′ > 150 ms, i.e., t′ = 200, 250, and 300 the amplitude of the maxFR for more preferred probes equated that of the reference alone, while for the less preferred it got closer to zero for late t′ followed by a smooth recovery with low but sustained firing.

In all cases the transient phases were followed by a recovery leading to a stationary rate. Since the sharp rebounding/dropping effect was a direct result of the presence of Ih and of the cell modulating its selectivity due to the probe being added, we hypothesize that as a result of trial and error such a change of concavity (inflection point in the first time derivative) may be utilized as a suitable selection cue to predict the stimulus' category. In particular, the computation of the instantaneous (not the average) derivative satisfies that requirement, and only demands local adaptation of the cell's firing.

### Comparing selectivity results in the model with experimental findings

**Figure 5:**
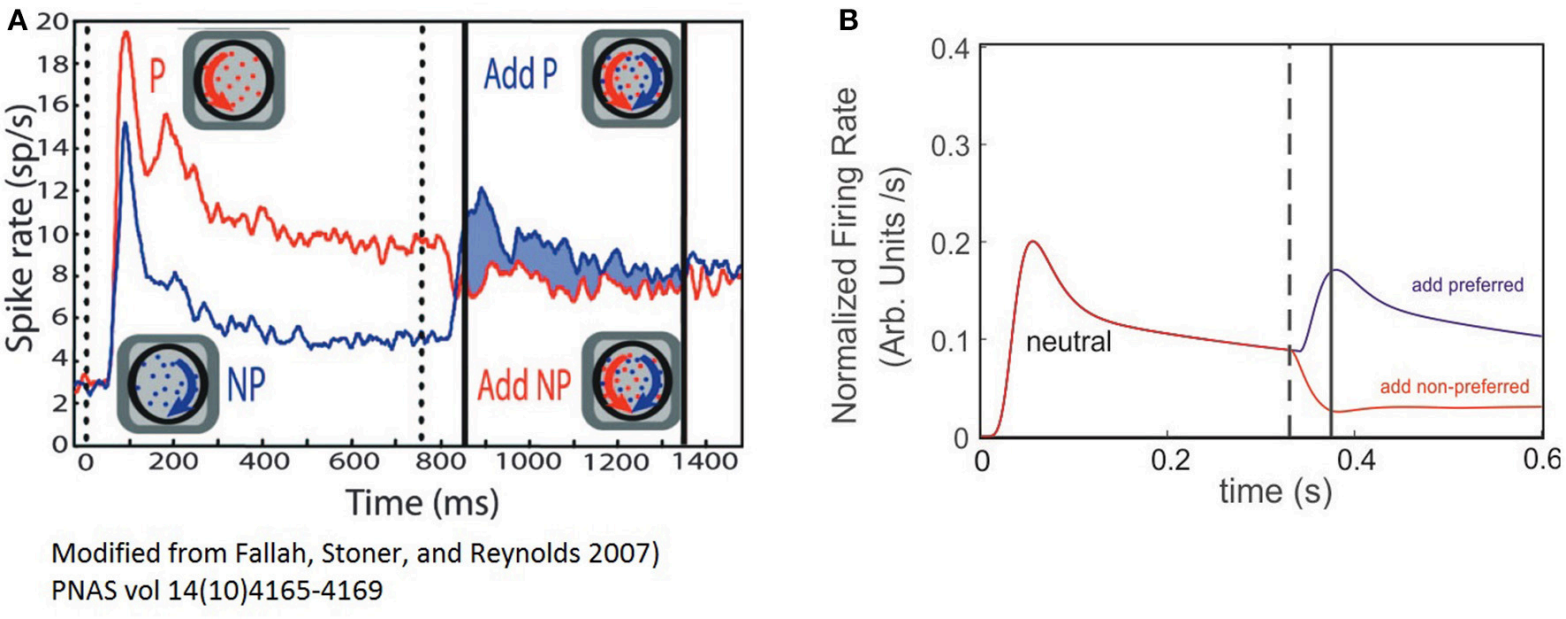
The ST model cell FR-profile reproduce experimental observations of V4 neurons. **(A)** Experimental firing rate computed on the population's activity of V4 neurons of primates, adapted from Fallah et al. (2007). The vertical dotted line indicates stimulus appearance, and the continuous black lines the period over which modulation of the response was computed. The red trace indicates the population response for the “preferred” (P) stimulus alone followed by the addition of the non-preferred (NP); while the blue trace indicates the non-preferred alone, followed by the addition of the preferred. **(B)** Simulated experiment. Both traces represent the response of the neuron when a neutral stimulus was presented followed by the addition of the preferred stimulus (red trace), or the non-preferred one (blue trace). The dashed line indicates the time at which the probe addition occurred and the continuous line the time of the transient's peak.

In a previous work on visual selection and color perception Fallah et al. (2007), measured the response of single neurons to a set of stimuli falling within its RF. Cells were located in the V4 extrastriate visual cortex in primates, and tuned to a particular hue. The animal was first exposed to a stimulus at *t* = 0, and at *t* = t′ a second with different hue structure was added. The recordings show a reshaping of the FR-profile in proportion to the relative match between the hue of the stimuli and the selectivity (selectivity) of the cell, producing FR patterns close to those shown in Figure 4, and depicted in Figure 5A. Ferrera et al. reported similar *in-vivo* dynamic while recording from cells in areas 7a, MT and V4 (Ferrera et al., 1994). Even though in both studies the outcome of the experiments clearly reflects correlations between the cell's response and feature-related information of the stimulus, the responsible mechanism was not characterized.

In order to explore the plausibility of the ST-cell dynamics with Ih in explaining those results, we implemented a high level simulation of Fallah's experiment using the circuit from Figure 1C The neutral reference (W_E−p_ = 0.7) was presented at *t* = 0 and a probe with larger or smaller selectivity was added at t′ = 300 ms. As a first confirmation of the model's efficacy, we observed that when starting with a neutral reference, the addition of more preferred probe (W_E−p_ = 0.80) induced a sharp increase in the FR and a bump with similar characteristics to the effects described in the previous section for probes with selectivity larger than the reference (compare blue traces in Figures 5A,B). In turn, a less preferred probe (W_E−p_ = 0.6) led to a drop and stabilization of the FR-profile (see red traces in Figures 5A,B).

In spite of the qualitative similarities between simulations and experiment, once the second stimulus is added, the experiment shows a brief period of non-responsiveness prior to a sharp modulation of firing which is underestimated in the model, but not necessarily as its flaw.

Since the biological problem suggests that for a particular combination of inputs, the neuron activation remains close to the resting state, the cell may react either by raising its firing, whenever the threshold is reached (generating a silent period of non-sensitive change), or by getting hyperpolarized and in consequence reducing the firing, which does not demand a threshold crossing and in consequence, no insensitive periods are required. Thus, we believe this is an aspect that needs further analysis and to account for the result, experiments using a broader range of selectivities need to be considered in a future study, together with further computational exploration.

### Effects of attention on the FR-profile

The most interesting aspect concerning the ST-characterization regards its behavior during attentional tasks. In this section we examine the extent to which attention could or not modulate the dynamics of the cell's selectivity.

As proposed by the Selective Tuning model (Tsotsos, 1990, 2011; Tsotsos et al., 1995), allocating/engaging attention in the model corresponds to the activation of the selection mechanism. Such mechanism was represented by a top down control signal responsible for suppressing information associated to irrelevant stimuli, while keeping unaffected the connections between the cells that processed information related to the attended stimulus in a task-dependent manner. We quantified the suppressive signal by computing the absolute difference between the weighted inputs impinging the top neuron, and used it to multiply the weight of the inputs from the unattended stimulus (see section Methods). This approach has proven to be fast and accurate at disambiguating stimuli, since rather than adding up the weighted contribution of all incoming signals, allows single neurons to efficiently filter them out and focus on the relevant ones. This idea is supported a key observation by Martinez-Trujillo et al., (Martinez-Trujillo and Treue, 2004; Khayat et al., 2010) according to which attention modulates the input to a given neuron instead of its direct response.

Using the circuit in Figure 1C, we studied the response of the top neuron when the reference and the probe were presented in isolation and simultaneously. In addition, to track possible variations in the stationary state, the attentional signal remained active until the end of the simulated period.

**Figure 6:**
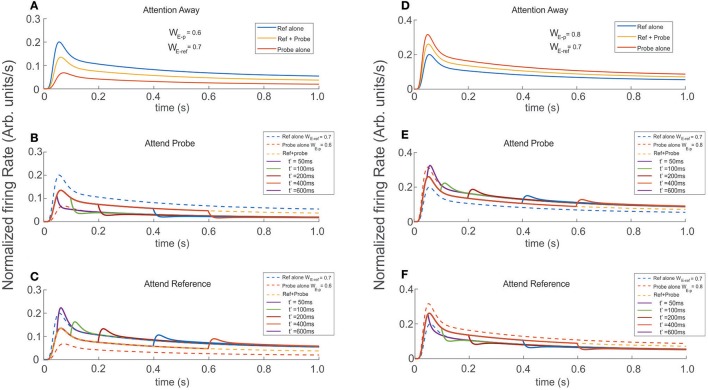
Engaging attention modulates the transients, and modifies the amplitude of the stationary response. Reference and probe stimuli were simultaneously presented and attended as indicated for each trace (see labels). The stimuli were presented at t = 0 ms and attention was engaged at t′ = 50, 100, 200, 400, 600 ms after stimulus presentation. **(A,D)** Show the reference and probe stimuli presented in isolation (blue and red traces) and simultaneously (yellow traces). In the first scenario, the reference is stronger and in the second the probe is stronger (higher selectivity). As expected the cell's selectivity mechanism produced firing rates with well differentiated maxFR's, each proportional to the respective selectivity of the stimulus. In addition, simultaneous reference and probe presentation, led to FR-profiles with intermediate amplitudes. Experiments were run for attention oriented to the probe **(B,E)**, and attention oriented to the reference **(C,F)**. Besides the characteristic transients, directing attention to the probe shifted the tail of the fr-baselines to the profile produced by the probe alone. Attending the reference produced similar effect on the fr-baseline, shifting in this case the tails of the response toward the reference alone FR-profile. Those long rate responses were consistent and irrespective of the relative selectivity between the reference and the probe.

In agreement with real experiments, and regardless of the amount of selectivity associated to each, when two stimuli of different selectivity were exposed to the scrutiny of the top neuron, the average behavior of the FR-profile fell in between those evoked by each stimulus in isolation; see Figures 6A,D. However, in the case of stimuli being simultaneously presented, a late engagement of attention to one of them modulated the cell's FR and forced it to adjust it to the magnitude evoked by the attended stimulus regardless of its selectivity, consistent with the theory (Martinez-Trujillo and Treue, 2004). The behavior is shown in Figures 6B,C, where the neutral reference (W_E−ref_ = 0.7) and the probe with less selectivity (W_E−p_ = 0.6) were both located inside the classical receptive field of the top neuron and simultaneously presented at t = 0 ms. When attention was allocated at t′ = 50, 100, 200, 400, and 600 ms, the FR rose or dropped accordingly to what stimulus was attended. Similar effects were obtained when the selectivity of the probe (W_E−p_ = 0.8) was larger than that of the reference, as shown in Figures 6E,F.

Irrespective of what stimuli was considered reference or probe, engaging attention to that of larger selectivity led the FR-profile to generate larger bumps (maxFR) than those observed for the attention away condition (dashed traces in Figures 6C,E); and FR with magnitude similar to the FR evoked by the largest stimulus in isolation. On the other hand, engaging attention to the stimulus with less selectivity produced FR-profiles characterized by troughs initiated at t′. In the case of Figure 6B the depth of the transient was more profound than in the case of the traces in Figure 6F, although in both cases the stationary response of the FR-profile coincided with that of the stimulus with less selectivity for the attention-away condition.

### Comparing the effect of attention in the ST-neuron with experimental recordings

**Figure 7:**
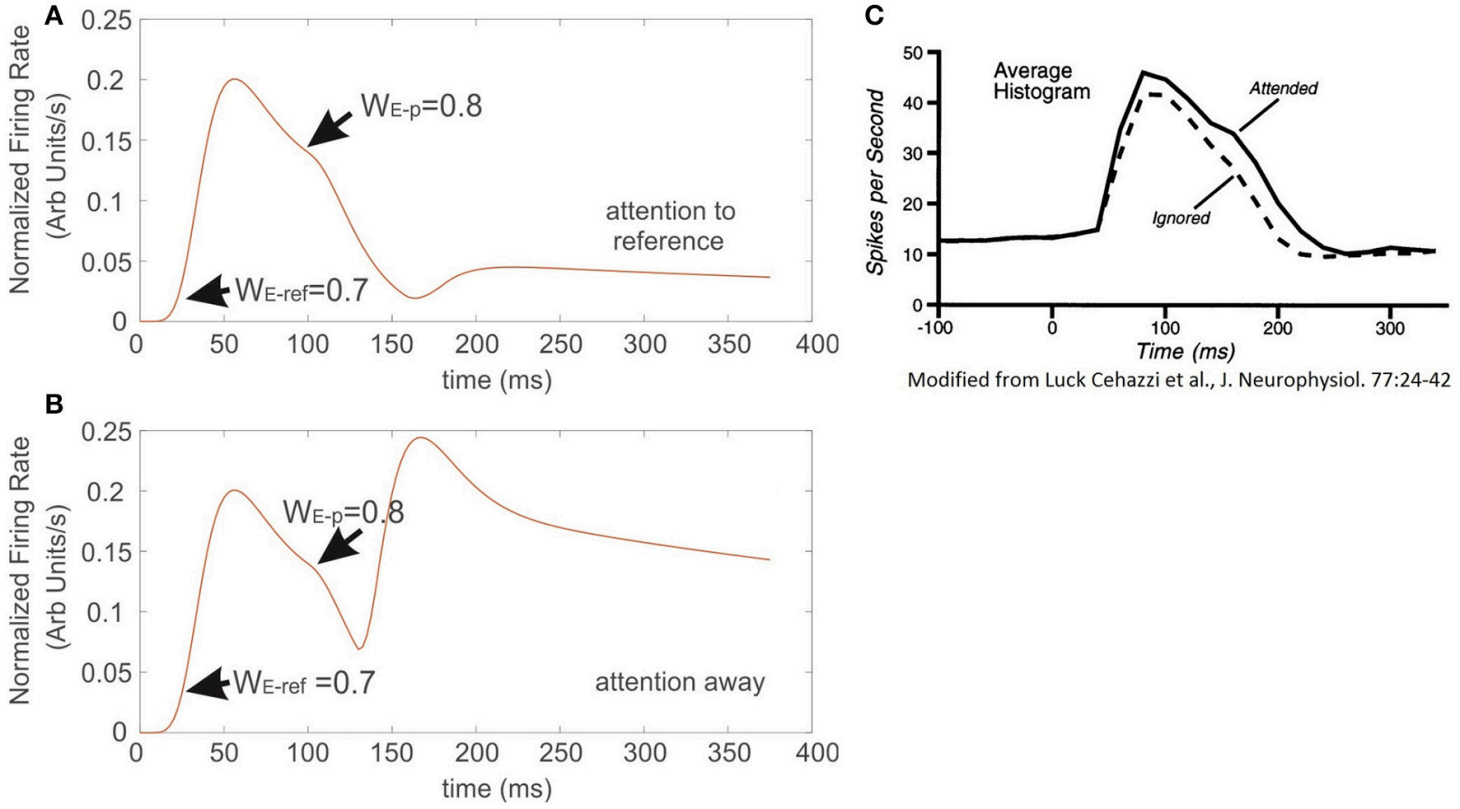
When attention competes against the effect of neural selectivity, it occludes the latter. Sequential presentation of stimuli with different selectivity inside the receptive field of the model neuron. **(A)** The reference stimulus is presented and attended at t = 0, once the stimulus switches to another with larger selectivity, but outside the still attended spot, the incoming stimulus barely altered the instantaneous firing of the cell, and only produced a negligible bump on the FR-profile at the exchange time. Condition **(B)** represents an identical setup to the one described in **(A)**, but in this case attention was not engaged to any of the stimuli. The absence of attention forced the firing of the cell to get immediately locked to the incoming input. The traces in **(C)** were adapted from Luck et al. (1997). They show that adding a probe with high selectivity to the receptive field of the top cell, while attending the reference also in the same receptive field, barely affects the ongoing response evoked by the reference.

Figures 7A,B correspond to the simulated conditions in which attention was either engaged to the reference with less selectivity (Figure 7A) or not allocated at all (Figure 7B). Interestingly, the resulting FR-profile in the first case shows a masking effect of attention that, in spite of a probe having larger selectivity than the reference, the FR gets modestly disrupted, remaining locked to the FR-profile of the reference. It contrasts the effect observed for the attention-away condition, in which the selectivity led the cell to rapidly increase the FR and adjust the FR-profile, matching that evoked by the probe alone, in this case with larger selectivity.

**Figure 8:**
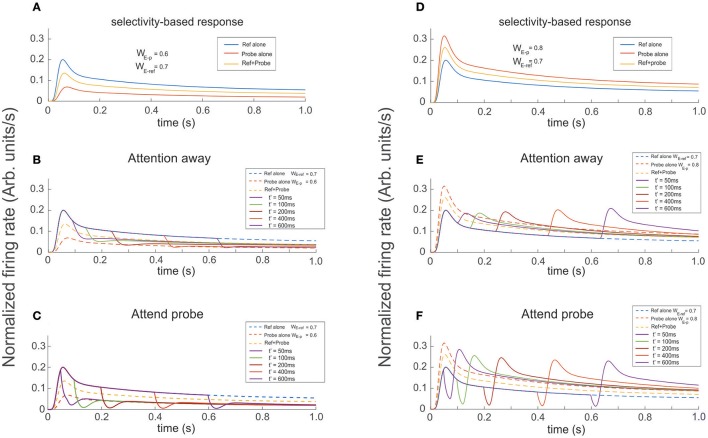
Presenting and attending a probe modulates the cell's selectivity effect. The reference was presented at *t* = 0 and the probe presented and attended as indicated for each color trace at t′ = 50, 100, 200, 400, 600 ms (see labels). **(A,D)** Show the firing rate for the stimuli presented in isolation (reference -blue, probe -red), and simultaneously (yellow trace). The three curves are also shown as dashed lines in **(B,C,E,F)** when the probe was added to the reference (both located inside the cell's receptive field) it has the effect to increase or reduce the cell's firing according to the cell's selectivity for the probe. In the attention away scenarios **(B,E)** the sole effect of selectivity, characterized by transients and baseline shifts, was observed. When Attention was engaged to the probe at t′, as shown in **(C,F)** it induced the occurrence of large transients with sharp changes of concavity, whose magnitude significantly depended on the respective cell's selectivity to the probe, relative to the response in the attention away scenarios. In addition, the magnitude of maxFR in the rebounding conditions were in average 30% larger, with slower decay times and tails shifting toward the FR-profile of the probe alone for more preferred probes, and toward the curve of the reference alone for probes with less selectivity, while in the attention away scenario the stationary response converged toward the profile evoked by both stimuli simultaneously presented.

In an experimental study Luck et al. (1997) measured single cell responses of neurons located at V4 associated to the appearance of a particular target. Stimuli were defined as *effective* or *ineffective* on a selectivity basis. In their protocol a series of trials consisted in presenting sequentially/simultaneously pairs of simple stimuli characterized by color and orientation, which could be both inside the cell's receptive field, or one inside and the other outside it, and attention was deployed to one of the two regions. For further details please refer to Luck et al. (1997). By comparing our results with those experimental recordings (Figures 7A,C respectively), the simulation shows good agreement, not only in the shape, but also in the time course of the FR-profile. In contrast to the condition observed in those figures, Figure 7B shows that in the absence of attention (attention away condition) there is no masking at all of the scene, and any probe stimulus with larger selectivity than the reference will draw the largest part of the cell response when both stimuli are located inside the RF. As in the experiment, Figure 7A shows the response of the top neuron after presenting the reference and probe simultaneously at *t* = 0, and the attentional mechanism is deployed at t = t′. Both simulation (Figure 7A) and experiment Figure 7C are characterized by a small modulatory dent in the cell's FR-profile while attending a less selective reference. The match between model and experiment suggests that in effect from the model's perspective, Ih makes the neuron highly sensitive to the effects of attention on selectivity (recall that in the absence of Ih the cell reached saturation, and the FR couldn't be modulated, see red trace in Figure 1A), but also from the biological perspective, the model suggests that attention and selection compete for resources when stimuli with low selectivity are attended. However, as it will be discussed later, the results in Figure 8 show that collaborative enhancement is also possible.

### Attention competes against or reinforces neural-selectivity

In our final experimental design, we ran simulations in which the reference was presented at *t* = 0 and the probe was presented and attended at t' = 50, 100, 200, 400, and 600 ms. Probes had either larger or smaller selectivity than the reference. In the attention away condition, a probe with less selectivity than the reference produced a decaying FR-profile characterized by shallow troughs and durations of the transient close to 150 ms, followed by a slow recovery of the FR in the direction of the stationary state (Figure 8D). In the same condition, probes with larger selectivity than the reference created rebounding firing rates with increasing amplitude, especially for late stimulus onset t′.

Running the same set of experiments while attention was allocated to the probe at t′ simultaneously with the probe's presentation, shows that attention has an ambiguous effect depending on whether the transient or the stationary dynamics of the cell's response were analyzed. As reference, Figures 8A,B show the FR-profile of the ST-model neuron in the attention away condition. All traces show that consistent with previous studies (Martinez-Trujillo and Treue, 2004), and based on its selectivity, the cell has larger maxFR for a more preferred stimulus and vice versa, while when the pair is active, the response always falls in between the FR-profile of the other two.

The effect of the selection mechanism of attention seemed to have a transitory component characterized by reinforcement of the cell's selectivity, while in the long term its behavior turned competitive. Although the affirmation may look contradictory, a careful check of Figures 8B,C shows that although the depth of the trough is larger for the attend-to-probe scenario, suggesting a steeper reduction of the FR (inhibition's reinforcement), the cell's response to the same onsets of the probe (indicated by traces of the same color in both figures) also corresponds to shorter widths (duration) of the trough in the attend-to-probe condition. In turn, when the FR was restored, the FR-profile matched that of the probe alone, in contrast to the attention-away condition (Figure 8B), in which the stationary state matched the FR-profile of the pair.

Interestingly, when a probe with larger selectivity than the reference was presented, it resulted in the opposite response of the neuron. A comparision between individual colored traces in Figures 8E,F shows that due to its large selectivity, a bump in the FR-profile occurred almost after the probe's onset in the attention away scenario, and that its magnitude increased by increasing the delay t′ between the onset of the reference and the probe, in a non-linear fashion (see bumps in Figure 8E). In the stationary state the solely effect of selectivity led the cell's FR-profile to match the response evoked by the pair.

In contrast, when the attentional mechanism was turned on while presenting the probe, the reduction in firing was represented by a deep and short trough characterizing the transitory response, exhibiting a duration of around 20 ms, similar for all t′, and depth with magnitude near to 20% of the maximum FR, except for t′ = 50 ms, (close to 30%).

This period that we called “latency,” preceded a bump in the FR-profile whose peak FR, was similar for most t′, and in general larger than the maxFR of the cell obtained when the pair was active, as shown in Figure 8F. Consistent with the case of the troughs, the peak of the bump for t′ = 50 ms was also slightly larger than for any other t′, suggesting that a short delay between the probe's onset and the activation of the attentional mechanism eases the processing of the stimulus of interest. Regarding the stationary response, we found the engagement of the FR to the response obtained when the probe was presented alone, in contrast to the attention away scenario, in which the FR was engaged to the FR-profile of the pair (see Figures 8E,F). It is important to note that in all simulations we implemented the selection mechanism of attention proposed by the ST model, which is based on inhibition of non-relevant inputs. In an earlier work by Busse et al. (2008), shifting attention from a cue located outside or inside of an MT cell's receptive to a probe in the opposite region was preceded by a drop in the firing rate of the cell. Authors claimed that the “short-latency decrease of responses” was caused by an interruption of endogenous attention, due to focusing on a stimulus that delayed the expected response toward the target.

**Figure 9:**
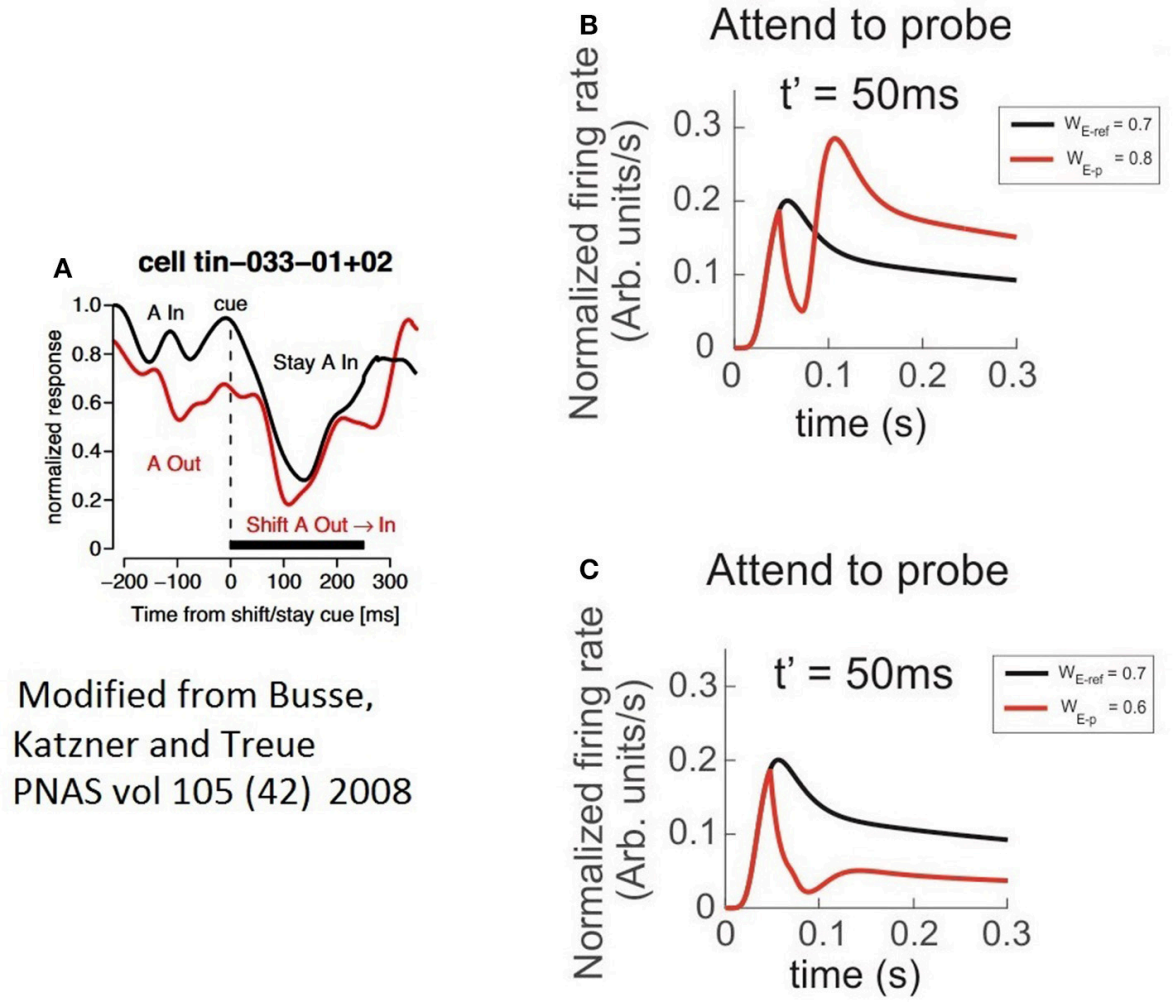
The characteristics of latencies preceding the attentional rebound depends on the relative selectivity between reference and probe. In the figure, a shift of attention occurs when a given cue (represented by a vertical dashed line) indicates the subject to attend outside of the receptive field of the measured neuron. **(A)** The red trace shows the neuron's response when the cue indicates the shift of the focus of attention from the outside to the inside, while the black trace reflects the response when the cue instructs attention to remain in the outside of the receptive field. Adapted from Busse et al. (2008). **(B)** Depicts the response of the top neuron (in the circuit of Figure 1C) to an initially non-attended neutral reference with onset at *t* = 0, while a preferred probe (W_E−ref_ = 0.7) is presented and attention is engaged at *t* = t′. In this case, the FR shows a short and sharp transient trough followed by a rebounding bump that engages the stationary FR-profile of the probe alone. **(C)** Represents a similar condition to **(B)** when using a less preferred probe than the reference. In spite of a significant reduction in the peak during the rebound, the stationary cell's response remains engaged to the FR-profile of the probe alone. The black traces in **(A,B)**, denote no shift of attention, cases in which the probe was absent and the reference remained in place until the end of the simulated period.

By restricting our analysis to the case in which attention switches from the outside to the stimulus in the inside (red trace in Figure 9A), similar to the Busse et al. experiment, our findings show a two-step process: first a drastic drop in the FR, and second, the steep recovery of firing that precedes a bump. It validates our observation that when a cell is initially active due to a cue with certain selectivity, attention leads the single cell's response to a brief interruption in the FR, represented by short and deep troughs in the FR-profile, regardless of the selectivity of a second stimulus; and to recover the FR following a time course whose shape (Figure 9A) is closely resembled by the model, as depicted by the red traces in Figures 9B,C. In our simulations the circuit in Figure 1C was initially exposed to the effect of a neutral reference (W_E−p_ = 0.7) and at t = t′ a probe with more/less selectivity was added to the cell's receptive field and attended. The model predicts a deeper trough for the preferred probe (W_E−p_ = 0.8) (Figure 9B) than for a non-preferred probe (W_E−p_ = 0.6) (Figure 9C), and both latencies having similar duration. However, additional experiments are required for a solid validation of this point. The study also suggests that the intention of switching attention generates a similar effect (black trace in Figure 9A), but because that there is no optimal way to simulate the intention of switching attention in the model, we represented that condition by leaving the reference stay during the whole simulation (see black traces in Figures 9B,C).

## Discussion

Attention is responsible for modulating the amount of input received by a neuron from the stimulus in its RF. In order to quantify the nature and magnitude of this modulatory effect, earlier studies (Pestilli et al., 2007) have reported significant correlation between attention and the dynamics of the threshold and contrast sensitivity processed single neurons, supporting some of their claims on the results of computational studies like the biased competition (Reynolds et al., 1999) and the multiplicative response gain model, that endow attention with an enhancment role of single neuron's activity (McAdams and Maunsell, 1999; Williford and Maunsell, 2006). In a theoretical study, Ladenbauer et al. (2014) presented a description of the effects of adaptation mechanisms, on the single cell's firing rate, highlighting a major influence on the gain of firing and threshold modulation, that agrees with the idea that external inhibitory synaptic inputs are relevant modulators of the input-output curve of single neurons.

A second intriguing element concerns the eventual generation of transients (bumps and troughs) in the firing rate of single cells (Martinez-Trujillo and Treue, 2004; Fallah et al., 2007; Busse et al., 2008), when a rapid stimulus switch takes place during attentional tasks, and that this particular response is due to suppression of irrelevant stimuli as previously posed by Lennert and Martinez-Trujillo (2011). In an earlier paper, Tsotsos (1990) first predicted such behavior, suggesting that inhibition of distractors allows the target neuron to restore its firing rate to the level evoked by the attended stimulus in isolation.

In this study we presented a revisited version of the ST neuron model, and characterized the effect on the firing rate of incorporating adaptation currents (Ih) into its dynamic equation, quantifying the neuron's response when submitted to various simulated experiments. We also strengthen the results of Rothenstein and Tsotsos (2014) describing the capabilities of the ST-neuron in reproducing experimental FR-profiles observed in simple attentional tasks, by separating the effects related to the cell's selectivity when Ih currents were active, from those related to attention. To our knowledge, this is the first time that adaptation current mechanisms are combined with an inhibition based model of the top-down attentive signal, to study the response of neurons in the visual cortex during attentive states.

With regard to the ST-neuron characterization, we found that in the absence of further mechanisms, the time course of the firing rate was driven by the balance between the constant σ of the Naka-Rushton term and the characteristic decay time of the inhibitory inputs. In turn, the modulation provided by Ih (depicted in Figures 1A, 2F) determined the existence of two regions in the FR-profile: the first quantifying the variability of the initial FR activation, and the second the post-saturation effect. Using a similar circuit to the originally proposed by Reynolds, we simulated the activation of V4 neurons, showing that selectivity creates a strong differentiation between patterns of response (FR-profiles), each possessing a unique maxFR (peak FR) and a stationary rate, correlated to the relevance of the input for the neuron. As an important aspect, the obtained FR-profile could be linked to different features of the stimulus or even to the whole stimulus (as in the case of V4 neurons) being represented not only by variations in the contrast or firing threshold.

The biological plausibility of the ST-neuron proved to be successful at reproducing different experimental scenarios, by only modulating the relation between inputs weights representing each stimulus. Our simulation of Fallah et al. experiment (Fallah et al., 2007), highlights the modulatory effect of Ih to reshape the FR, when responding to stimuli with significant differences in the selectivity in the absence of attention. Although the model predicts changes in the transitory state of the FR, further experiments are required to verify the prediction.

The significance of the Ih dynamics proved its relevance also in more complex scenarios that included activation of the attentional signal. As described in Results, we showed that by incorporating the selection mechanism of attention proposed by Tsotsos (1990), the FR-profile resembled the response of real V4 neurons, and that by using Reynold's design (Figure 1C), as seen in Figure 7. A no enhancement is necessary to account for the time course of the firing rate when stimuli with different levels of neural selectivity are presented in isolation or simultaneously. Furthermore, our simulations show that by including the activation of the attentional mechanism, the FR was able to differentially represent possible conditions for the onsets of attention, or its shift in a non-redundant way, for different experimental designs, regardless of how similar can be the stimuli. In this scenario we show the interplay between selectivity and attention (Figures 6A,B) is crucial to define the dynamics of the FR when two stimuli suddenly switch with each other, affecting both the transitory and the stationary phases of the FR-profile. We predict the existence of a dual role played by attention, in which it can enhance or compete against selectivity during the transitory stage, and the opposite during the stationary stage, depending on how preferred each stimulus is for the neuron. The plausibility of our results is strongly backed up by the significant resemblance obtained by simulating the Luck et al. (1997), and Busse et al., experiments (Busse et al., 2008), in which the change of selectivity in the first (Figure 7C) together with the deployment of attention, and the shift of the focus of attention in the second (Figure 9), are well accounted by the significant changes in both phases of the FR-profile. Overall, the behavior of the ST-model reflects the context-based competitive or enhancing effect of the cross-talk between attention and selectivity.

Our results coincided with the claim posed by the ST-model (Tsotsos, 1990; Tsotsos and Rothenstein, 2011) that suppressing irrelevant activity in the surround of the attentional focus forces the cell to adapt its firing and match the rate evoked by the attended stimulus in isolation, in the sense that when attended, the FR-profile of the neuron in all simulations depended on its selectivity to that stimulus regardless of stimulus context. It made the response produced by all stimuli within the receptive field to be larger in the unattended scenario than when one of them was attended, due to the presence of distractors with high selectivity in the surrounding.

Since a significant amount of the information was encoded by the transient (latency), we hypothesize that this period of average duration in the range 20–30 ms, during which the firing rate suddenly drops and raises, could be required for the cells to re-accommodate to the confluent and ongoing bottom-up effect of selectivity and the top-down signal of attention; however, future work will require experiments in single cells and populations to test the functioning principles of the latency periods, so as to characterize their time courses. Secondly, based on our hypotheses it will be necessary to also check if the interplay between attention and selectivity is enough to fully disambiguate stimuli with complex combinations of features within a single visual scene.

## Author contributions

This research work was carried out in collaboration between all authors. OA and JT defined the research theme. OA and JT designed methods and simulations, OA analyzed the data, OA and JT interpreted the results and wrote the paper. OA and JT discussed analyses, interpretation, and data presentation. All authors have contributed to, seen and approved the manuscript.

### Conflict of interest statement

The authors declare that the research was conducted in the absence of any commercial or financial relationships that could be construed as a potential conflict of interest.
